# Development and Application of Genomic Control Methods for Genome-Wide Association Studies Using Non-Additive Models

**DOI:** 10.1371/journal.pone.0081431

**Published:** 2013-12-16

**Authors:** Yakov A. Tsepilov, Janina S. Ried, Konstantin Strauch, Harald Grallert, Cornelia M. van Duijn, Tatiana I. Axenovich, Yurii S. Aulchenko

**Affiliations:** 1 Institute of Cytology and Genetics SD RAS, Novosibirsk, Russia; 2 Novosibirsk State University, Novosibirsk, Russia; 3 Institute of Genetic Epidemiology, Helmholtz Zentrum München - German Research Center for Environmental Health, Neuherberg, Germany; 4 Institute of Medical Informatics, Biometry and Epidemiology, Chair of Genetic Epidemiology, Ludwig-Maximilians-Universität, Munich, Germany; 5 Research Unit of Molecular Epidemiology, Helmholtz Zentrum München - German Research Center for Environmental Health, Neuherberg, Germany; 6 Department of Epidemiology, Erasmus MC Rotterdam, Netherlands; 7 Centre for Population Health Sciences, University of Edinburgh, Edinburgh, United Kingdom; The University of Chicago, United States of America

## Abstract

Genome-wide association studies (GWAS) comprise a powerful tool for mapping genes of complex traits. However, an inflation of the test statistic can occur because of population substructure or cryptic relatedness, which could cause spurious associations. If information on a large number of genetic markers is available, adjusting the analysis results by using the method of genomic control (GC) is possible. GC was originally proposed to correct the Cochran-Armitage additive trend test. For non-additive models, correction has been shown to depend on allele frequencies. Therefore, usage of GC is limited to situations where allele frequencies of null markers and candidate markers are matched.

In this work, we extended the capabilities of the GC method for non-additive models, which allows us to use null markers with arbitrary allele frequencies for GC. Analytical expressions for the inflation of a test statistic describing its dependency on allele frequency and several population parameters were obtained for recessive, dominant, and over-dominant models of inheritance. We proposed a method to estimate these required population parameters. Furthermore, we suggested a GC method based on approximation of the correction coefficient by a polynomial of allele frequency and described procedures to correct the genotypic (two degrees of freedom) test for cases when the model of inheritance is unknown. Statistical properties of the described methods were investigated using simulated and real data. We demonstrated that all considered methods were effective in controlling type 1 error in the presence of genetic substructure. The proposed GC methods can be applied to statistical tests for GWAS with various models of inheritance. All methods developed and tested in this work were implemented using R language as a part of the GenABEL package.

## Introduction

Genome-wide association studies (GWAS) are a powerful tool for mapping genes of complex traits. Standard statistical methods used for GWAS, such as linear regression, assume that the correlation between a phenotype and a genotypic marker exists because of the marker itself or a strong linkage disequilibrium with the causative locus. This assumption holds when the sample consists of representatives of one panmictic population. However, other correlations caused by confounding factors that influence both phenotypes and genotypes of various loci are possible. In GWAS, the genetic substructure of the studied samples is among the most important confounders. If the analysis is not accounted for confounding by population substructure, the test statistic is inflated [1], which makes its statistical interpretation difficult and may lead to false-positive findings.

If information on a large number of genetic markers is available, the analysis results can be adjusted by accounting for the influence of non-specific effects by using the genomic control (GC) method. Several methods have been proposed for GC adjustment [1]–[5]. Devlin and Roeder [1] suggested the use of a correction coefficient, denoted as variance inflation factor (VIF), to correct the distribution of the test statistic. In general, the VIF has been demonstrated to be a function of marker allele frequencies and population parameters [1]. It has also been deduced that for an additive model, the VIF does not depend on allele frequency. Thus, for an additive model, the “GC inflation factor” constant, λ, can be empirically estimated from null (not associated) loci. Note, however, that for smaller allele frequencies and smaller samples asymptotic assumptions will not hold, and, consequently, the inflation of the test statistic will depend on allele frequencies even for additive model.

Several estimators of the Genomic Control inflation constant λ could be used. For example, the mean test statistic is an estimator of λ, which, however, suffers from being strongly affected by outliers (e.g., from true association signals). The median estimator (*λ_median_*), which is defined as ratio of the median of the observed distribution of the test statistic and 0.455 (the median of the 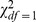 distribution) [1], is probably the one used most. Another estimator can be defined as regression coefficient of the observed test statistic onto the statistic expected for the null loci (regression estimator *λ_regress_*). This estimator arises from the simple observation that the covariance between two ordered random variables one of which is distributed as 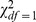 and another as λ*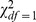 is equal to 2*λ, while the variance of the expected distribution is 2. All of these estimators are constants that we can use as indicators of statistical bias or as coefficients allowing correction of the observed test statistic.

The general formulation of the VIF [2], in principle, allows for extension of GC to dominant and recessive models. However, for the non-additive model, the VIF depends on allele frequency and a number of parameters that describe the genetic structure of the sample. Thereby, it is possible to estimate the VIF empirically (as for additive model) if the allele frequency of null loci is the same as for the test locus (specific VIF for each allele group), but in this case the number of available null markers is limited and thus limits the applicability of the GC method. An alternative way requires estimation of the population structure parameters. The methods, which infer population structure and assign individuals to populations [6] are computationally extensive.

Another method for empirical VIF estimation was suggested by Zheng et al. [3] for .a two degrees of freedom (2df) model, which does not constrain the relation of phenotypes and genotypes and does not impose severe restrictions on the weight of the heterozygous genotype. This “robust GC” method was based on combining the corrected test statistics from dominant and recessive models [3]. Yet another method of correction – delta decentralization (based on centralization of the non-central chi-square) – was proposed by Gorroochurn et al [7], but was later invalidated by Dadd et. al. [8].

In this work, we aimed to develop and evaluate existing methods for GC correction of results of GWAS using non-additive (recessive, dominant, over-dominant, and 2df genotypic) models. Therefore, we concentrate on several points: formulation of VIF expressions for various models with one degree of freedom (1df) and development of VIF-based procedures for GC correction of the results of these models; estimation of model parameters describing the population substructure for VIF estimation; development of a new “polynomial” GC (PGC) method based on a polynomial approximation of the correction coefficient that can be applied for both one- and two-degree tests. All methods were tested using simulated and real data.

## Results

### VIF for non-additive models

We derived the VIF as function of allele frequency (*p*), model of inheritance (*x* indicates the effect of the heterozygous genotype; for recessive, additive, and dominant model, *x* is equal to 0, 0.5, and 1, respectively), sample size (*N*), and population parameters. The over-dominant model (effect of genotype is equal to 0 for homozygotes and to 1 for heterozygotes) is described separately. Population parameters include the Wright's coefficient of inbreeding *F* (ranging from 0 to 1) [9] and a coefficient that describes the population substructure, 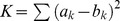 (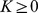), where 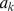 and 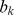 are numbers of representatives of each of the subpopulations in case and control samples, respectively. In reality, the mean inbreeding *F* takes a values of <0.01 for most populations, but can reach values of 0.04 for highly consanguineous populations [10]. The value of *K/N^2^* approaches zero when the design is balanced (e.g. case:control ratio is 1∶1 in each subpopulation) and approaches its maximum of 1/2 when either cases or controls only are sampled from each subpopulation.

The VIF is obtained as 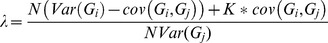, where *G_i_* is the marker genotype of the *i*-th case (*G_i_*∈ {0,1,2}). 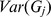 and 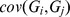 is defined as:
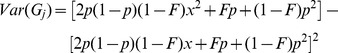
and
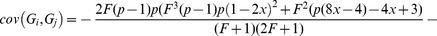


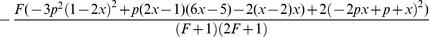
respectively. The derivations and detailed formulas for the VIF are provided in the Supplementary Note S1.

**Figure 1:**
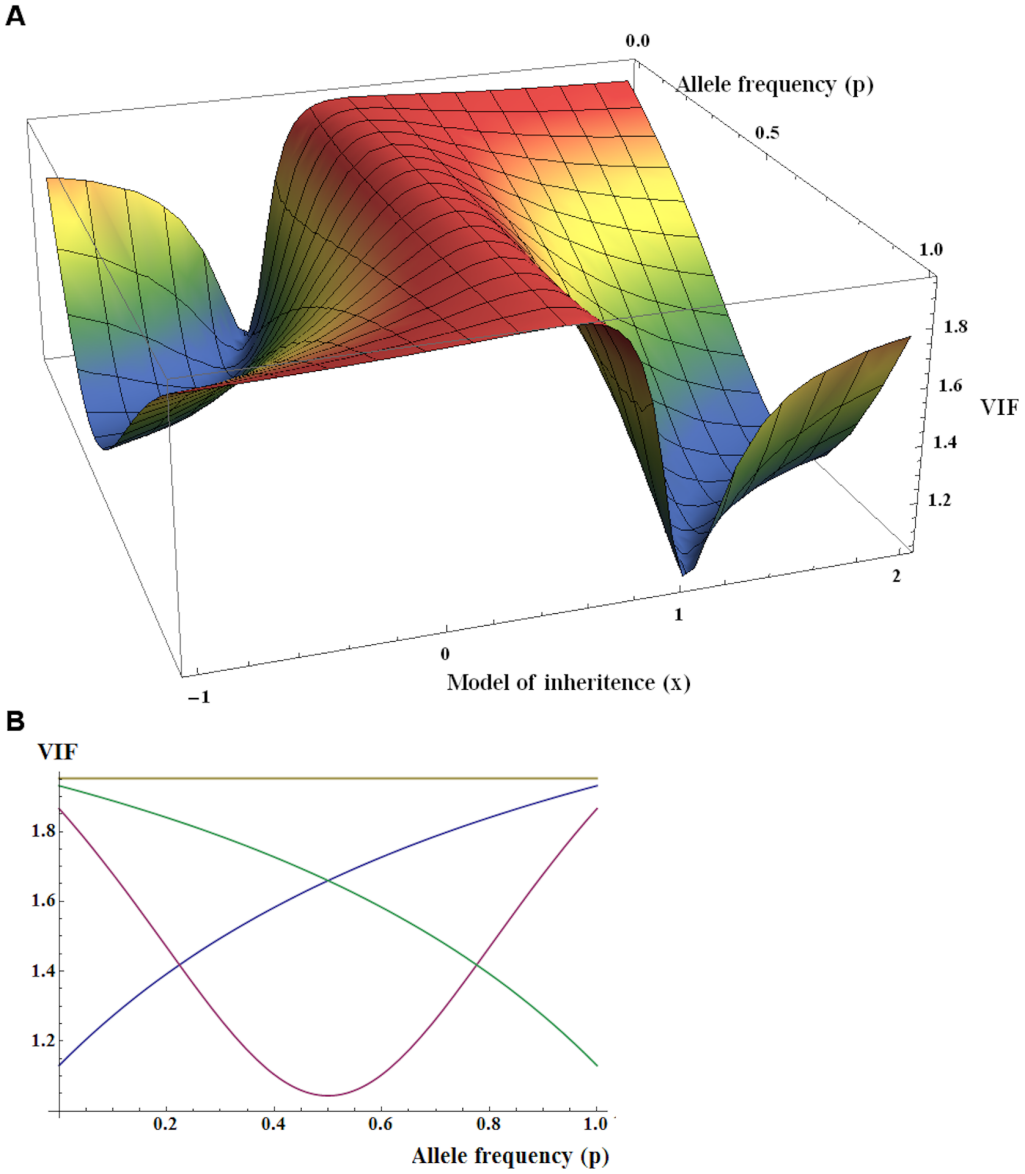
Dependence of VIF function on allele frequency *p* and model parameter *x* (*F* = 0.05; *N* = 1,000; *K* = 11,000). (A) *p*: {0,1}, *x*: {−1,2}; (B) *p*: {0,1}, *x = *0 (R, recessive), *x = *1 (D, dominant), *x = *1/2 (A, additive), *x = *100 (O, over-dominant).

Figure 1 presents the VIF function for a set of population parameters (*F* = 0.05; *N* = 1,000; *K* = 11,000). This figure shows that the VIF is independent of allele frequency only for the additive model (*x* = ½), which has been demonstrated previously [2]. The function is point symmetric at *x = *½ - recessive model is mirror image of dominant. Also for *x* tending to infinity, it approaches – as expected – the function for the over-dominant model of inheritance.

Several conclusions could be drawn from the analysis of the VIF function (Figure 1). First, the VIF of an additive model is always greater than the one of non-additive models, which we also observed in the analysis of simulated data for uncorrected tests (Table 1). Second, the application of a *naive* correction by a constant to the results obtained from the non-additive model GWAS can fix the “average” type 1 error to the nominal level; however, for several markers, the test will be conservative and for others, it will be liberal. For example, for the dominant model, such a ”correction” will lead to a liberal test for low frequency SNPs (single nucleotide polymorphisms) and to a conservative test for common SNPs. These results are confirmed by our simulations for constant correction tests (Table 1). Although the correction by a constant generally keeps the type 1 error rate to a pre-defined threshold, this is not true for SNPs in a particular frequency group.

**Table 1:** Type 1 error for one degree of freedom tests.

		Not corrected			Constant corrected			VIFGC corrected			PGC corrected		
Model	Frequency	*λ_median_*	*λ_regress_*	*E*	*λ_median_*	*λ_regress_*	*E*	*λ_median_*	*λ_regress_*	*E*	*λ_median_*	*λ_regress_*	*E*
**Reccessive**	all	1.301	1.305	0.086	1.000	1.003	0.051	1.000	1.000	0.050	0.999	0.999	0.050
	[0.05,0.25)	1.175	1.170	0.069	0.905	0.900	0.038	0.990	0.983	0.048	1.004	0.998	0.049
	[0.25,0.4)	1.245	1.245	0.079	0.957	0.957	0.045	0.995	0.995	0.049	1.000	1.000	0.050
	[0.4,0.6)	1.320	1.322	0.088	1.014	1.015	0.052	1.002	1.004	0.051	0.998	0.999	0.050
	[0.6,0.75)	1.377	1.381	0.095	1.057	1.060	0.057	1.006	1.009	0.051	0.996	0.999	0.050
	[0.75,0.95]	1.412	1.416	0.100	1.084	1.087	0.060	1.007	1.010	0.051	0.997	1.000	0.050
**Additive**	all	1.453	1.458	0.104	1.000	1.003	0.051	0.997	1.000	0.050	0.991	1.034	0.050
	[0.05,0.25)	1.451	1.455	0.104	0.998	1.001	0.050	0.995	0.998	0.050	0.991	1.033	0.050
	[0.25,0.4)	1.455	1.460	0.105	1.001	1.005	0.051	0.998	1.002	0.050	0.991	1.035	0.050
	[0.4,0.6)	1.456	1.461	0.105	1.002	1.006	0.051	0.999	1.002	0.051	0.990	1.035	0.050
	[0.6,0.75)	1.454	1.458	0.104	1.000	1.003	0.051	0.997	1.000	0.050	0.990	1.034	0.050
	[0.75,0.95]	1.452	1.456	0.104	0.999	1.002	0.051	0.996	0.998	0.050	0.992	1.036	0.050
**Dominant**	all	1.302	1.306	0.086	1.000	1.003	0.051	0.999	1.000	0.050	0.999	1.000	0.050
	[0.05,0.25)	1.413	1.416	0.099	1.084	1.086	0.060	1.007	1.009	0.051	0.997	0.999	0.050
	[0.25,0.4)	1.379	1.383	0.095	1.058	1.061	0.057	1.007	1.010	0.051	0.997	1.000	0.050
	[0.4,0.6)	1.320	1.323	0.088	1.013	1.016	0.052	1.002	1.004	0.051	0.998	1.001	0.050
	[0.6,0.75)	1.244	1.245	0.079	0.956	0.956	0.045	0.993	0.993	0.049	1.000	1.000	0.050
	[0.75,0.95]	1.174	1.171	0.070	0.903	0.900	0.039	0.988	0.984	0.048	1.003	0.999	0.050
**Over-dominant**	all	1.176	1.181	0.072	1.000	1.004	0.051	0.999	1.000	0.050	0.999	1.000	0.050
	[0.05,0.25)	1.281	1.282	0.083	1.088	1.089	0.061	1.007	1.008	0.051	0.996	0.997	0.050
	[0.25,0.4)	1.143	1.146	0.067	0.972	0.974	0.047	0.998	1.000	0.050	1.006	1.008	0.051
	[0.4,0.6)	1.060	1.058	0.057	0.902	0.901	0.039	0.987	0.985	0.048	0.991	0.990	0.049
	[0.6,0.75)	1.142	1.143	0.067	0.971	0.972	0.047	0.998	0.999	0.050	1.006	1.007	0.051
	[0.75,0.95]	1.279	1.282	0.083	1.086	1.089	0.060	1.007	1.008	0.051	0.996	0.997	0.050

Type 1 error was estimated in three ways: *λ_median_*, which is the ratio of observed distribution's median and expected median; *λ_regress_*, which is the regression coefficient between observed statistic's distribution and theoretically expected Chi-square statistic; and *E*, which is the proportion of the tests with p-value≤0.05. The values are given for all SNPs as well as for stratified frequency groups.

### Estimation of VIF parameters

Methods for VIF estimation require knowledge about parameters that describe the population substructure. If these parameters, such as *F* and *K*, are not known, estimates can be utilized. Estimation is based on the idea that the distribution of the analysis test statistic should follow 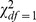 after correction. Thereby, estimating unknown function parameters is possible by minimizing a chosen error function that indicates the deviation of the observed distribution from the expected one. We used a sum of squared deviations of the ordered corrected statistics (*Z^2^*) and the theoretically expected distribution as an error function:
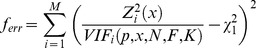



Note that only the population parameters *F* and *K* should be estimated, whereas *N* (sample size), *M* (number of SNPs), and *p* and *x* are defined by the data and the analysis model. This method was denoted as VIFGC.

### Polynomial GC

We also propose a polynomial GC for non-additive models, which approximates the correction function via an *l-*degree polynomial of allele frequency *p*:
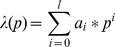



For estimation of the coefficients 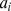, we used the same idea as with the estimation of parameters *F* and *K* in method VIFGC, that is that the corrected statistic 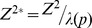 should be distributed as 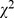. We denote this method as PGC. Empirically, we decided to use third degree polynomials during the optimization.

These two refined strategies, VIFGC and PGC, were compared with the standard GC method of dividing test statistics by a constant λ. We used simulated and real data to evaluate the type 1 error and power of the methods. Type 1 error was characterized in three ways: *λ_median_*, which is the ratio of the observed distribution's median and the expected median (0.455); *λ_regress_*, which is the regression coefficient between the observed statistic's distribution and the theoretically expected 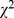 statistic; and *E*, which is the proportion of tests with p-value ≤ nominal level. We also characterized the type 1 error in five specific marker allele frequency groups: [0.05,0.25), [0.25,0.4), [0.4,0.6), [0.6,0.75), and [0.75,0.95].

### Simulation results

Simulation details could be found in the “Materials and methods section”. Simulation results for type 1 error for 1df tests are presented in Table 1. As discussed above, constant corrected tests have significant deviations from expected values of type 1 error for several allele frequency groups for non-additive models. Unlike the constant correction method, the PGC and VIFGC methods have a type 1 error close to expected values both for all SNPs together and for specific frequency groups.

Simulation results for type 1 error for 2df tests are presented in Table 2. This table shows that constant corrected tests have slightly liberal type 1 error with a behavior similar to the additive model, and the inflation does not depend on allele frequencies. The method based on 1df VIFGC-corrected tests is strongly conservative (the type 1 error is lower than the nominal level). PGC corrected tests have type 1 error levels close to the nominal level.

**Table 2:** Type 1 error for two degrees of freedom tests.

	Not corrected			Constant corrected			df1 based (VIFGC corrected)*			PGC corrected		
Frequency	*λ_median_*	*λ_regress_*	*E*	*λ_median_*	*λ_regress_*	*E*	*λ_median_*	*λ_regress_*	*E*	*λ_median_*	*λ_regress_*	*E*
**all**	1.239	1.250	0.092	1.000	1.009	0.053	0.951	0.957	0.045	0.991	1.000	0.051
**[0.05,0.25)**	1.239	1.248	0.091	1.000	1.007	0.052	0.959	0.962	0.045	0.992	1.000	0.051
**[0.25,0.4)**	1.241	1.252	0.092	1.001	1.010	0.053	0.948	0.955	0.045	0.991	1.000	0.051
**[0.4,0.6)**	1.240	1.252	0.092	1.001	1.010	0.053	0.942	0.951	0.044	0.990	1.000	0.051
**[0.6,0.75)**	1.239	1.251	0.092	1.000	1.009	0.053	0.946	0.955	0.045	0.990	1.000	0.052
**[0.75,0.95]**	1.239	1.249	0.092	1.000	1.008	0.053	0.959	0.963	0.045	0.992	1.000	0.051

The abbreviations are as in Table 1. The values are given for all SNPs as well as for stratified frequency groups.

* 2df test based on 1df corrected tests (here, 1df tests were corrected by VIFGC) [3].

The power of different methods is shown in Table 3. It showed that all methods for correction including VIFGC and PGC have optimal power when the correct model (the one used in the simulations) is also used for analysis. As expected, the 2df genotypic test has less power but is robust compared with the model used for simulation.

**Table 3:** Power (% of test with p-value≤0.05) for different tests.

Simulated model	Recessive					Additive					Dominant					Over-dominant				
Analised model	r	a	d	o	g	r	a	d	o	g	r	a	d	o	g	r	a	d	o	g
**Not corrected**	0.87	0.71	0.26	0.44	0.78	0.60	0.78	0.64	0.42	0.67	0.20	0.74	0.84	0.39	0.78	0.37	0.32	0.40	0.83	0.76
**Constant corrected**	0.79	0.59	0.15	0.43	0.64	0.48	0.67	0.58	0.38	0.62	0.15	0.63	0.80	0.35	0.72	0.31	0.26	0.33	0.78	0.60
**VIF corrected** *	0.80	0.59	0.16	0.41	0.62	0.50	0.66	0.55	0.38	0.58	0.15	0.63	0.80	0.35	0.68	0.30	0.26	0.32	0.77	0.57
**PGC corrected**	0.81	0.58	0.16	0.41	0.63	0.50	0.67	0.56	0.38	0.62	0.15	0.64	0.80	0.36	0.72	0.30	0.26	0.32	0.77	0.59

* genotypic model for VIFGC corrected tests is a two degrees of freedom test based on recessive and dominant tests corrected by VIFGC [3].

r, a, d, o, and g are recessive, additive, dominant, over-dominant, and genotypic models, respectively.

### Application to real data

Real data application on two independent cohorts, namely, Cooperative Health Research in the region of Augsburg (KORA) and Erasmus Rucphen Family (ERF), provided the opportunity to test our methods in situations that are not reflected in our simulation study. In both studies, we analyzed imputed genotypes (expressed as estimated probabilities) and quantitative traits using linear regression methods. While our previous derivations and results concern binary traits, an important previous observation for the GC was that the method can be applied in the framework of quantitative trait regression analysis models as well [11].

It should be noted, that for genome-wide analyses in ERF, mixed model based methods are used [12]; here, however, we use ERF as an example of highly genetically structured population, and analyze it using fixed effects-only model. In KORA, we analyzed uric acid levels, and in ERF, levels of high-density lipoprotein (HDL) (see “Materials and Methods” section for more details).

Table 4 shows the results of type 1 error analysis in ERF, a family-based study where the association is strongly confounded by the genetic structure if *naïve* analysis is applied. When an additive model is used without correcting for genetic structure via mixed models, λ for HDL is 1.2. For non-additive models, we reproduced the same principal findings that we obtained from simulated data by using these real data. Correction by a constant factor results in a conservative test for some frequency groups and in a liberal test for other frequency groups, whereas VIFGC and PGC corrections yield accurate levels independent of the marker allele frequency.

**Table 4:** Type 1 error in ERF data analysis.

		Not corrected			Constant corrected			VIFGC corrected*			PGC corrected		
Model	Frequency	*λ_median_*	*λ_regress_*	*E*	*λ_median_*	*λ_regress_*	*E*	*λ_median_*	*λ_regress_*	*E*	*λ_median_*	*λ_regress_*	*E*
**Recessive**	all	1.201	1.200	0.074	1.000	1.000	0.050	1.001	1.000	0.050	1.000	1.000	0.050
	[0.05,0.25)	1.105	1.109	0.063	0.921	0.924	0.042	0.993	0.997	0.050	0.998	1.002	0.051
	[0.25,0.5)	1.188	1.180	0.072	0.989	0.983	0.048	1.005	0.999	0.050	0.998	0.992	0.050
	[0.5,0.75)	1.240	1.252	0.079	1.033	1.043	0.055	1.004	1.013	0.051	0.996	1.006	0.051
	[0.75,0.95]	1.273	1.261	0.081	1.061	1.051	0.056	1.001	0.991	0.049	1.008	0.999	0.050
**Additive**	all	1.298	1.302	0.086	1.000	1.003	0.051	0.997	1.000	0.050	0.996	1.000	0.050
	[0.05,0.25)	1.299	1.290	0.085	1.001	0.994	0.050	0.998	0.991	0.049	1.008	1.000	0.050
	[0.25,0.5)	1.298	1.313	0.088	1.001	1.012	0.052	0.997	1.008	0.052	0.986	0.997	0.050
	[0.5,0.75)	1.301	1.320	0.088	1.003	1.017	0.053	1.000	1.014	0.052	0.989	1.003	0.051
	[0.75,0.95]	1.292	1.286	0.084	0.995	0.991	0.049	0.992	0.988	0.049	1.003	0.999	0.050
**Dominant**	all	1.202	1.203	0.074	1.000	1.001	0.050	1.000	1.000	0.050	1.000	1.000	0.050
	[0.05,0.25)	1.277	1.265	0.081	1.063	1.053	0.056	1.001	0.991	0.049	1.008	0.999	0.050
	[0.25,0.5)	1.243	1.252	0.079	1.035	1.042	0.054	1.003	1.011	0.051	0.997	1.005	0.051
	[0.5,0.75)	1.190	1.183	0.072	0.990	0.985	0.049	1.005	1.000	0.050	0.999	0.994	0.050
	[0.75,0.95]	1.104	1.112	0.064	0.919	0.925	0.042	0.991	0.998	0.050	0.995	1.002	0.051
**Over-dominant**	all	1.133	1.123	0.064	1.000	0.991	0.049	1.011	1.000	0.050	1.011	1.000	0.050
	[0.05,0.25)	1.203	1.193	0.072	1.061	1.053	0.056	1.017	1.010	0.051	1.011	1.003	0.050
	[0.25,0.5)	1.076	1.060	0.057	0.950	0.935	0.043	1.011	0.995	0.049	1.013	0.998	0.049
	[0.5,0.75)	1.064	1.053	0.056	0.939	0.929	0.042	1.000	0.989	0.049	1.004	0.993	0.049
	[0.75,0.95]	1.204	1.190	0.072	1.062	1.050	0.056	1.017	1.007	0.051	1.014	1.004	0.051
**Genotypic (df = 2)**	all	1.157	1.162	0.077	1.000	1.004	0.052	0.964	0.966	0.046	0.996	1.000	0.051
	[0.05,0.25)	1.163	1.159	0.077	1.005	1.002	0.052	0.973	0.969	0.047	1.004	1.001	0.051
	[0.25,0.5)	1.152	1.165	0.077	0.996	1.006	0.052	0.954	0.964	0.045	0.988	0.999	0.051
	[0.5,0.75)	1.151	1.164	0.077	0.995	1.006	0.052	0.953	0.963	0.046	0.989	1.000	0.051
	[0.75,0.95]	1.163	1.159	0.077	1.005	1.001	0.052	0.973	0.970	0.047	1.004	1.000	0.052

* for VIFGC corrected genotypic (2df) tests, we used the 1df based test by performing VIFGC-corrected tests for recessive and dominant models [3].

The abbreviations are as in Table 1. The values are given for all SNPs as well as for stratified frequency groups.

Type 1 error results in KORA, a population-based study where stratification is minimal, are presented in Table 5. When an additive model is used, we observe that λ is only 1.03 for the quantitative trait uric acid. For non-additive models, where the genetic structure is close to absent in this data set, we still reproduced the same principal findings as observed in our simulations and analysis of ERF data.

**Table 5:** Type 1 error of KORA data tests.

		Not corrected			Constant corrected			VIFGC corrected*			PGC corrected		
Model	Frequency	*λ_median_*	*λ_regress_*	*E*	*λ_median_*	*λ_regress_*	*E*	*λ_median_*	*λ_regress_*	*E*	*λ_median_*	*λ_regress_*	*E*
**Recessive**	all	1.016	1.020	0.053	1.000	1.004	0.051	0.996	1.000	0.050	0.996	1.000	0.050
	[0.05,0.25)	1.015	1.016	0.052	0.998	1.000	0.050	1.004	1.005	0.051	1.000	1.001	0.050
	[0.25,0.5)	1.014	1.023	0.053	0.998	1.006	0.051	0.996	1.005	0.051	0.992	1.001	0.050
	[0.5,0.75)	1.017	1.021	0.053	1.001	1.005	0.051	0.993	0.997	0.050	0.993	0.997	0.050
	[0.75,0.95]	1.020	1.020	0.053	1.004	1.004	0.051	0.993	0.993	0.050	1.000	1.001	0.051
**Additive**	all	1.019	1.024	0.053	1.000	1.005	0.051	0.995	1.000	0.050	0.995	1.000	0.050
	[0.05,0.25)	1.024	1.030	0.053	1.005	1.011	0.051	1.000	1.006	0.051	0.997	1.003	0.050
	[0.25,0.5)	1.020	1.024	0.053	1.001	1.004	0.051	0.996	0.999	0.050	0.994	0.998	0.050
	[0.5,0.75)	1.017	1.019	0.052	0.998	1.000	0.050	0.993	0.995	0.050	0.994	0.996	0.050
	[0.75,0.95]	1.016	1.024	0.053	0.997	1.005	0.051	0.992	1.000	0.050	0.995	1.003	0.051
**Dominant**	all	1.021	1.025	0.053	1.000	1.004	0.051	0.996	1.000	0.050	0.996	1.000	0.050
	[0.05,0.25)	1.024	1.026	0.053	1.002	1.005	0.051	0.989	0.992	0.049	0.999	1.002	0.050
	[0.25,0.5)	1.025	1.028	0.054	1.004	1.007	0.051	0.995	0.999	0.050	0.994	0.998	0.050
	[0.5,0.75)	1.015	1.026	0.053	0.994	1.005	0.051	0.992	1.003	0.050	0.987	0.997	0.050
	[0.75,0.95]	1.022	1.021	0.053	1.000	1.000	0.050	1.007	1.007	0.051	1.003	1.002	0.050
**Over-dominant**	all	1.027	1.027	0.053	1.000	1.000	0.050	1.000	1.000	0.050	1.000	1.000	0.050
	[0.05,0.25)	1.027	1.027	0.054	1.000	1.001	0.051	0.987	0.988	0.049	0.999	1.000	0.050
	[0.25,0.5)	1.029	1.027	0.053	1.003	1.000	0.050	1.016	1.013	0.051	1.003	1.001	0.050
	[0.5,0.75)	1.028	1.025	0.053	1.002	0.998	0.050	1.014	1.011	0.051	1.002	0.999	0.050
	[0.75,0.95]	1.021	1.028	0.054	0.995	1.001	0.051	0.982	0.988	0.049	0.994	1.000	0.051
**Genotypic (df = 2)**	all	1.021	1.024	0.054	1.000	1.003	0.051	0.999	1.002	0.051	0.997	1.000	0.051
	[0.05,0.25)	1.021	1.022	0.054	1.000	1.001	0.051	0.997	0.998	0.050	1.000	1.001	0.051
	[0.25,0.5)	1.022	1.026	0.055	1.001	1.005	0.051	0.997	1.001	0.051	0.996	1.000	0.051
	[0.5,0.75)	1.020	1.024	0.054	0.999	1.003	0.051	0.996	0.999	0.050	0.994	0.998	0.050
	[0.75,0.95]	1.021	1.025	0.055	1.000	1.004	0.052	1.006	1.009	0.052	0.997	1.001	0.051

* for VIFGC corrected genotypic (2df) tests, we used the 1df based test by performing VIFGC-corrected tests for recessive and dominant models [3].

The abbreviations are as in Table 1. The values are given for all SNPs as well as for stratified frequency groups.

## Discussion

We demonstrated by simulations and the analysis of real data that the proposed GC methods (VIFGC and PGC) could be used for the correction of non-additive test statistics in the context of GWAS assuming different models of inheritance. For additive models, widely used in GWAS, there are two applications of GC methods. Firstly, the inflation coefficient λ can be used to correct the test statistic, thereby making an interpretation of *p*-values statistically valid. Secondly, λ serves as an important indicator of goodness of the model used for association analysis. Although no specific threshold is available, as a rule, if the inflation of the test statistics is relatively large, this reflects the fact that the model chosen for analysis poorly accounts for the genetic structure present in the sample. In that case, the analysis model should be revised, e.g., instead of standard linear regression, the use of a such methods as structured association, EIGENSTRAT [13] or mixed models [14]–[16] should be considered. Note that even after the most advanced analysis model is used, some residual inflation may be expected. This residual inflation is usually corrected by the GC, because even minor inflation still can lead to much increased false positive rate in GWAS. For example, at λ = 1.05, when the test statistic is not corrected, the *χ^2^* threshold of 29.72 (p-value = 5*10^−8^ in case the statistic is not inflated) corresponds to p-value = 1*10^−7^, that is the false positive rate is increased by more than two times. This correction is also very important when meta-analysis of multiple GWAS is performed [17] because a small residual inflation, when not corrected, can lead to very large inflation in the final meta-analysis test statistic.

In our examples involving analysis of real phenotypes, the main use of GC in ERF is the use as indicator. Although nominal type I error can be achieved with the GC, GWAS should be performed with mixed models in this population, and GC should be used only to correct residual inflation. Analysis of KORA, which is a carefully designed population-based study with little stratification, provides a more realistic example of a case when the GC method should be used “directly”.

Most GWAS performed up-to-date have used an additive model, and GC is an essential part of the analysis procedure. Methods for GC for non-additive models are much less developed. This obstructs correct analysis, meta-analysis, and interpretation of the results of such GWAS. Despite weaker development of the methodological base, some works reported interesting findings based on the analysis of non-additive models [18]–[21].

In this work, we proposed new and study existing methods of GC for non-additive models. We demonstrated that the VIFGC and the PGC method can be used to correct the results of GWAS obtained by using dominant, recessive, over-dominant (one degree of freedom), and genotypic (two degrees of freedom) models. We show that in general, for 1df models, both VIFGC and PGC perform equally well, whereas for the 2df model, the test based on a 1df VIFGC-corrected statistic results in a conservative test. Thus, for the genotypic model, the PGC correction may be preferred. These methods have a variance of the type 1 error in all frequency groups that is significantly less than that observed when constant correction GC is applied (*p*<10^−20^, see Table S4). It should be noted that for the PGC method we could in principle use an exponential function instead of a polynomial, but using of exponential function restricts the available models to the recessive and dominant only. Using polynomial functions eliminates restrictions for using PGC for other models, such as overdominant and the 2df genotypic ones.

However, the quality of approximation comes at the costs of time whereas the correction of the additive model's results is computationally very simple, the VIFGC and PGC corrections include parameter optimization steps. Still, even PGC that requires optimization of four parameters and uses the data from 2.5 million tests finishes within minutes on a standard PC.

In our methods, we estimated the parameters by using the regression loss function. This loss function is sensitive to possible heavy tails of the distribution (these may reflect real strong association signals). Therefore, we decided to use the lower 95% of the distribution, which is similar to the method suggested in [22]. Other solutions are possible by using different loss functions, which are less sensitive to outliers. Examples include loss functions defined by the sum of absolute deviations or the square root thereof and the difference between obtained and expected medians.

For additive models, the GC inflation factor λ is an important indicator of goodness of the model used for association analysis. For recessive, dominant, and over-dominant models, we have demonstrated that the test inflation is always smaller than the inflation for the additive model (Figure 1). Therefore, we suggest that the use of analytical method that appropriately reflects the genetic structure of the underlying data should be decided based on λ for an additive model or based on the maximal value of the VIF function from the non-additive model.

For the GC method, an important previous observation was that the method is not specific for the Cochran-Armitage test. Bacanu et. al. [11] have demonstrated earlier that the GC method can be applied in the framework of quantitative trait regression analysis models as well, including models with gene-gene interaction. We confirmed this principle by analyzing real quantitative trait in two different populations.

All methods developed and tested in this work were implemented in R language in the GenABEL-package [23], part of the GenABEL project for statistical genomics (http://www.genabel.org).

In summary, we proposed and tested several methods for GC for various models of inheritance and compared these methods by using real and simulated data. We demonstrated that the VIFGC and the PGC method can be successfully used in adjusting the test statistic for different non-additive models in the framework of GWAS.

## Materials and Methods

### ERF study

Simulations were based on real genetic data collected in the framework of the Erasmus Rucphen Family (ERF) study. All study protocols were approved by the Medical Ethics Committee of Erasmus University, and all participants gave written informed consent in accordance with the Declaration of Helsinki. The ERF study is a cross-sectional study embedded in genetically isolated population located in southwest Netherlands. The study participants are members of a single large pedigree that can be traced in 23 generations and contains thousands of cycles [24]. The sample used for simulations included 3,235 people, for whom the genotypes of 54,000 SNP markers were available. All SNPs included had a coding allele frequency (CAF) 0.05≤CAF (coded allele frequency) ≤0.95 and a call rate ≥0.95.

In the example where real phenotypic data was used, we analyzed levels of high-density lipoprotein (HDL) on imputed genotypic data. This data set included 2,699 people, who were genotyped and imputed (using HapMap2 as reference panel) at 2,093,818 SNP markers. All SNPs in the subset had 0.05≤CAF≤0.95 and a call rate ≥0.95. More detailed description of phenotyping and sample can be found in [25].

### KORA study

As an example of a carefully designed population-based study, we used KORA (Cooperative Health Research in the region of Augsburg) F4, which is a study from the KORA cohorts [26]. KORA F4 is a follow-up (from 2006 to 2008) of the population-based KORA S4 study that was conducted in the region of Augsburg in Southern Germany from 1999 to 2001. All study protocols were approved by the ethics committee of the Bavarian Medical Chamber (Bayerische Landesärztekammer), and all participants gave written informed consent. In our application, we analyzed levels of uric acids in a data set including 1,788 people who were genotyped with the Affymetrix 6.0 SNP array (730,525 SNP markers after quality control) with further imputation using HapMap2 (release 22) as reference panel resulting in a total of 2,210,193 SNPs. All SNPs in the study had 0.05≤CAF≤0.95 and a call rate ≥0.95. A more detailed description of study design, genotyping, and phenotyping is reported in [27].

### Simulations

The phenotypes for analysis of type 1 error and the power were simulated based on real genetic data from the ERF study by using the scheme described below. We used binary traits for simulations because in our own derivation of VIF as well as in previous derivations, binary traits were used in the same way as demonstrated in [2]. Results can be generalized to quantitative traits as well [11].

Liability values were simulated as a sum of independent quantitative trait loci (QTLs) and polygenic effects. The heritability coefficient was set to be equal to a random number coming from a uniform distribution bounded by 0.5 and 0.8. To model the QTL effect, an SNP was randomly chosen. Based on its minor allele frequency (MAF), the effect was assigned in a way that the SNP was accounted for 0% of total liability variance for type 1 error and 0.35% for power simulations. To model the polygenic effect, 500 markers were randomly chosen (excluding the chromosome harboring the QTL), and based on their allele frequencies, effects were assigned in such way that each of the SNPs explained the same fraction of non-QTL heritability. The quantitative phenotype was transformed into a binary trait following a threshold model (the “case” phenotype was assigned if liability was below the threshold corresponding to 1/3 of the distribution). To study type 1 error, 1,000 simulation cycles were performed. To study the power, 100 simulation cycles were performed.

### Association analysis

For the analysis of simulated and real data, we used standard tests implemented in the GWFGLS (genome-wide feasible generalized least squares) function of the MixABEL package, which is a part of the GenABEL suite of programs [23] for statistical genomics (option “score,” so the output from GWFGLS for binary traits was completely the same as for Cochran-Armitage trend test of chi-square for binary traits). GWAS were calculated for five different (additive, dominant, recessive, over-dominant, and genotypic) models of SNP effect.

For quantitative trait analysis, we used regression and score test as implemented in MixABEL. For the analysis of imputed data, the regression was performed onto probabilities.

GWAS results were corrected using different methods for GC. The standard method, which corrects test statistic by dividing it by the estimated λ, was applied as well as two refined methods. The qualities of the GC correction methods were compared with one another in terms of type 1 error with three characteristics:

*λ_median_*: ratio of distribution's median and expected median (0.455)

*λ_regress_*: regression coefficient between statistic's distribution and theoretically expected Chi-square statistic

*E*: proportion of the tests with p-value less then declared level (0.05).

In addition to the comparison of the performance for all SNPs, five allele frequency groups were compared separately: [0.05,0.25), [0.25,0.4), [0.4,0.6), [0.6,0.75), and [0.75,0.95].

## Supporting Information

Note S1 **Derivation of VIF for non-additive models.**
(DOC)

Table S1 **The distribution of genotypes of biallelic markers in the “case-control” design.**
(DOC)

Table S2
**Formalization of the 1df models of inheritance using different genotype coding** 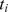 **.**
(DOC)

Table S3 **Joint probability distribution of genotype frequencies.**
(DOC)

Table S4 **Results** of Levene's test for homogeneity of variance between two correction methods of E* (proportion of the tests with p-value≤0.05) - in simulations for type 1 error. Var1 and Var2 – total variance of E* in all frequency groups for the first and second method, respectively. Ratio – ratio of Var1 and Var2.
(DOC)

## References

[1] Devlin B, Roeder K (1999) Genomic control for association studies. Biometrics 55: 997–1004. 11315092 10.1111/j.0006-341x.1999.00997.x

[2] Zheng G, Freidlin B, Li Z, Gastwirth JL (2005) Genomic control for association studies under various genetic models. Biometrics 61: 186–192. 15737092 10.1111/j.0006-341X.2005.t01-1-.x

[3] Zheng G, Freidlin B, Gastwirth JL (2006) Robust genomic control for association studies. American journal of human genetics 78: 350–356. 16400614 10.1086/500054 PMC1380242

[4] Zang Y, Zhang H, Yang Y, Zheng G (2007) Robust genomic control and robust delta centralization tests for case-control association studies. Human heredity 63: 187–195. 17310128 10.1159/000099831

[5] Yan T, Hou B, Yang Y (2009) Correcting for cryptic relatedness by a regression-based genomic control method. BMC genetics 10: 78. 19954543 10.1186/1471-2156-10-78 PMC3087514

[6] Pritchard JK, Stephens M, Donnelly P (2000) Inference of population structure using multilocus genotype data. Genetics 155: 945–959. 10835412 10.1093/genetics/155.2.945 PMC1461096

[7] Gorroochurn P, Heiman GA, Hodge SE, Greenberg DA (2006) Centralizing the non-central chi-square: A new method to correct for population stratification in genetic case-control association studies. Genetic epidemiology 30: 277–289 10.1002/gepi.20143 16502404

[8] Dadd T, Lewis CM, Weale ME (2010) Delta-centralization fails to control for population stratification in genetic association studies. Human heredity 69: 285–294 10.1159/000302720 20389097

[9] Oliehoek PA, Windig JJ, van Arendonk JAM, Bijma P (2006) Estimating relatedness between individuals in general populations with a focus on their use in conservation programs. Genetics 173: 483–496 10.1534/genetics.105.049940 16510792 PMC1461426

[10] Bittles A (2001) Consanguinity and its relevance to clinical genetics. Clinical genetics 60: 89–98. 11553039 10.1034/j.1399-0004.2001.600201.x

[11] Bacanu S-A, Devlin B, Roeder K (2002) Association studies for quantitative traits in structured populations. Genetic epidemiology 22: 78–93. 11754475 10.1002/gepi.1045

[12] Aulchenko YS, Struchalin M V, van Duijn CM (2010) ProbABEL package for genome-wide association analysis of imputed data. BMC bioinformatics 11: 134. 20233392 10.1186/1471-2105-11-134 PMC2846909

[13] Price AL, Patterson NJ, Plenge RM, Weinblatt ME, Shadick NA, et al (2006) Principal components analysis corrects for stratification in genome-wide association studies. Nature genetics 38: 904–909. 16862161 10.1038/ng1847

[14] Yu J, Pressoir G, Briggs WH, Vroh Bi I, Yamasaki M, et al (2006) A unified mixed-model method for association mapping that accounts for multiple levels of relatedness. Nature genetics 38: 203–208. 16380716 10.1038/ng1702

[15] Chen W-M, Abecasis GR (2007) Family-based association tests for genomewide association scans. American journal of human genetics 81: 913–926. 17924335 10.1086/521580 PMC2265659

[16] Dupuis J, Langenberg C, Prokopenko I, Saxena R, Soranzo N, et al (2010) New genetic loci implicated in fasting glucose homeostasis and their impact on type 2 diabetes risk. Nature genetics 42: 105–116. 20081858 10.1038/ng.520 PMC3018764

[17] De Bakker PIW, Ferreira MAR, Jia X, Neale BM, Raychaudhuri S, et al (2008) Practical aspects of imputation-driven meta-analysis of genome-wide association studies. Human molecular genetics 17: R122–8. 18852200 10.1093/hmg/ddn288 PMC2782358

[18] Wellcome Trust Case Control Consortium (2007) Genome-wide association study of 14,000 cases of seven common diseases and 3,000 shared controls. Nature 447: 661–678 10.1038/nature05911 17554300 PMC2719288

[19] Liu J, Xiao Q, Wang Y, Xu Z-M, Yang Q, et al (2013) Analysis of genome-wide association study-linked loci in Parkinson's disease of Mainland China. Movement disorders: official journal of the Movement Disorder Society 10.1002/mds.25599 23853107

[20] Kobayashi D, Nishizawa D, Takasaki Y, Kasai S, Kakizawa T, et al (2013) Genome-wide association study of sensory disturbances in the inferior alveolar nerve after bilateral sagittal split ramus osteotomy. Molecular pain 9: 34 10.1186/1744-8069-9-34 23834954 PMC3723511

[21] McLaren PJ, Coulonges C, Ripke S, van den Berg L, Buchbinder S, et al (2013) Association Study of Common Genetic Variants and HIV-1 Acquisition in 6,300 Infected Cases and 7,200 Controls. PLoS pathogens 9: e1003515 10.1371/journal.ppat.1003515 23935489 PMC3723635

[22] De Bakker PIW, Raychaudhuri S (2012) Interrogating the major histocompatibility complex with high-throughput genomics. Human molecular genetics 21: R29–36. 22976473 10.1093/hmg/dds384 PMC3459647

[23] Aulchenko YS, Ripke S, Isaacs A, van Duijn CM (2007) GenABEL: an R library for genome-wide association analysis. Bioinformatics (Oxford, England) 23: 1294–1296. 10.1093/bioinformatics/btm108 17384015

[24] Liu F, Ikram MA, Janssens ACJW, Schuur M, de Koning I, et al (2009) A study of the SORL1 gene in Alzheimer's disease and cognitive function. Journal of Alzheimer’s disease: JAD 18: 51–64. 19584446 10.3233/JAD-2009-1137

[25] Aulchenko YS, Ripatti S, Lindqvist I, Boomsma D, Heid IM, et al (2009) Loci influencing lipid levels and coronary heart disease risk in 16 European population cohorts. Nature genetics 41: 47–55. 19060911 10.1038/ng.269 PMC2687074

[26] Wichmann H-E, Gieger C, Illig T (2005) KORA-gen–resource for population genetics, controls and a broad spectrum of disease phenotypes. Gesundheitswesen (Bundesverband der Ärzte des Öffentlichen Gesundheitsdienstes (Germany)) 67 (Suppl 1) S26–30. 16032514 10.1055/s-2005-858226

[27] Kolz M, Johnson T, Sanna S, Teumer A, Vitart V, et al (2009) Meta-analysis of 28,141 individuals identifies common variants within five new loci that influence uric acid concentrations. PLoS genetics 5: e1000504. 19503597 10.1371/journal.pgen.1000504 PMC2683940

