# Supplementary material for: Development and Application of Genomic Control Methods for Genome-Wide Association Studies Using Non-Additive Models
Source: PLoS One. 2013 Dec 16;8(12):e81431. doi: 10.1371/journal.pone.0081431 (PMC3864791; doi:10.1371/journal.pone.0081431)
Supplement: Note S1 — Derivation of VIF for non-additive models. (DOC) [file pone.0081431.s001.doc]

# Supplementary note S1

The “Сase-control” design of experiment is commonly used for GWAS of binary traits.

For each marker locus in each group the number of people with each of the possible genotypes is counted (Table S1).

For formalizing one degree of freedom (1df) models of inheritance the effect of genotype $t_{i}$ is indicated, where *i* denotes the genotype index (0, 1, 2 for *a_1_a_1_, a_1_a_2_, a_2_a_2_*, correspondently). For the additive, recessive and dominant models it is accepted that the effects of genotypes *a_1_a_1_* and *a_2_a_2_* are 0 and 1, correspondently, and the effect of the genotype *a_1_a_2_* depends on the model. (Table S2).

The Cochran-Armitage statistic can be used for testing these models. It takes into account the dosage of influence factor (the effect of genotype $t_{i}$) in the two groups. This statistic tests the hypothesis about equal distribution of the genotype frequencies in the two groups using the model of inheritance (the more the effect of genotype $t_{i}$, the more difference of the weight of the genotype frequencies). It is computed as *Z^2^ = T^2^/Var (T)* and its large sample distribution is approximated by a chi-square with 1df. The values of *T* and *Var(T)* are considered by the formulas:

$$T=\sum_{i=0}^{2} t_{i}(r_{i}S-s_{i}R)$$

$$Var\left(T\right)=\frac{SR}{N}(\sum_{i=0}^{2} t_{i}^{2}m_{i}(N-m_{i})-2\sum_{i=0}^{1} \sum_{j=i+1}^{2} t_{i}t_{j}m_{i}m_{j})$$

The expression for the score test for specified model of inheritance *x* is given by:

$$Z_{x}^{2}=\frac{N[N\left(xr_{1}+r_{2}\right)-R(xm_{1}+m_{2})]}{R\left(N-R\right)[\left(x^{2}m_{1}+m_{2}\right)-(xm_{1}+m_{2})^{2}]}$$

As stated earlier, the distribution in a real situation, as a rule, is not described by the central chi-square distribution due to genetic structuring of the sample. In view of this, we have proposed the following algorithm to calculate the correction factor for various models of inheritance.

Denote by *G_i_*∈ {0,1,2}, *i* = 1,...,R the marker genotype of the *i*-th case, by *H_j_*, *j* = 1,...,S the genotype of the *j*-th control from the sample. The Cochran-Armitage statistic Z^2^ is proportional to the square of the statistic T, defined differently as

$$T=\sum_{i=1}^{R} G_{i}-\sum_{j=1}^{S} H_{j}$$

In general form, the variance of *T* can be written as

$$Var\left(T\right)=\sum_{i=1}^{R} Var(G_{j})+\sum_{j=1}^{S} Var(H_{j})+2\sum_{i<l} cov\left(G_{i},G_{l}\right)+2\sum_{j<l} cov\left(H_{j},H_{l}\right)-2\sum_{i} \sum_{j} cov\left(G_{i},H_{j}\right)$$

Under the null hypothesis of no association between the marker and the disease, expected value *E(T)=0*, variance $Var(G_{j})=Var(H_{j})$ and $cov\left(G_{i},G_{l}\right)= cov\left(H_{j},H_{l}\right)= cov\left(G_{i},H_{j}\right)$, so

$Var_{0}\left(T\right)=NVar(G_{j})-Ncov\left(G_{i},G_{j}\right)$ (1.1)

When studied individuals selected from a homogeneous population of unrelated individuals, under the null hypothesis:

$Var_{0}\left(T\right)=NVar(G_{j})$ (1.2)

It is easy to show that under Hardy-Weinberg equilibrium:

$Var(G_{j})=(2pqx^{2}+p^{2})-(2pqx+p^{2})^{2}$ (1.3)

where *p* is the frequency of allele *a_2_* in the population, and *q = 1-p*.

If the Hardy-Weinberg equilibrium is disturbed, for example, because of related structure or subpopulations, it is known that increased frequencies of homozygous genotypes are:

$$\Pr\left(AA\right)=Fp+(1-F)p^{2}$$

$$\Pr\left(Aa\right)=2\left(1-F\right)pq$$

$$\Pr\left(aa\right)=Fq+(1-F)q^{2}$$

Here *F* – Wright's inbreeding coefficient.

In this case, $Var(G_{j})$ takes the form:

$Var(G_{j})=\left[2pq(1-F)x^{2}+Fp+(1-F)p^{2}\right]-[2pq(1-F)x+Fp+(1-F)p^{2}]^{2}$ or

$Var\left(G_{i}\right)=p(2(F-1)(p-1)x^{2}-p(2(F-1)(p-1)x+F(-p)+F+p)^{2}-Fp+F+p)$ (1.4)

Furthermore, F ≠ 0 implies covariance between members of this population. To evaluate them, we must first estimate the joint distribution of genotypes in a pair of individuals, which was done in [2] (Table S3).

If the genotype frequencies of the pairs of individuals are known (Table S3), the covariance between the effects of genotypes in these individuals is

$cov\left(G_{i},G_{j}\right)=[\Pr\left(AA,AA\right)-(\Pr\left(AA\right))^{2}]+2x\left[Pr\left(AA,Aa\right)-2\Pr\left(AA\right)\Pr\left(Aa\right)\right]+$ $+x^{2}[\Pr\left(Aa,Aa\right)-(\Pr\left(Aa\right))^{2}]$ (1.5)

or

$cov\left(G_{i},G_{j}\right)=\Pr\left(AA,AA\right)-(Fp+(1-F)p^{2})^{2}+2\left[xPr\left(AA,Aa\right)-4xpq\left(Fp+\left(1-F\right)p^{2}\right)\left(1-F\right)\right]+$ $+x^{2}[\Pr\left(Aa,Aa\right)-(2\left(1-F\right)pq)^{2}]$ (1.6)

Substituting all the values from the table 3 we obtain the following:

$$cov\left(G_{i},G_{j}\right)=-\frac{2F(p-1)p(F^{3}(p-1)p\left(1-2x\right)^{2}+F^{2}(p(8x-4)-4x+3)}{(F+1)(2F+1)}-$$

$-\frac{F(-3p^{2}\left(1-2x\right)^{2}+p(2x-1)(6x-5)-2(x-2)x)+2\left(-2px+p+x\right)^{2})}{\left(F+1\right)\left(2F+1\right)}$ (1.7)

Suppose that the sample consists of the representatives of *m* subpopulations. The number of representatives of each of the subpopulations in patients denoted as *а_1_, а_2_,...,a_m_*, and in the sample of controls as *b_1_, b_2_,...,b_m_*. It is logical to assume that the covariance will be observed only within a subset of, but not among different subpopulations. It was shown earlier [2] that:

$Var\left(T\right)=NVar\left(G_{i}\right)+cov(G_{i},G_{j})\sum_{k} \left\{a_{k}\left(a_{k}-1\right)+b_{k}\left(b_{k}-1\right)-2a_{k}b_{k}\right\}$ (1.8)

or

$Var\left(T\right)=N\left(Var\left(G_{i}\right)-cov\left(G_{i},G_{j}\right)\right)+cov\left(G_{i},G_{j}\right)\sum^{ } (a_{k}-b_{k})^{2}$ (1.9)

here *k* = 1,...,*m*.

Variance inflation factor (VIF) is denoted as

$\lambda=\frac{Var(T)}{Var_{0}(T)}$ (1.10)

where $Var\left(T\right)$ and $Var_{0}(T)$ defined by (1.9) and (1.2), respectively.

After substituting *x* values as 0, 0.5, 1 we obtained expressions derived earlier in [2,5].

For the overdominant model, the expressions were obtained similarly, except the weights of genotypes taken 0, 1, 0 for *a_1_a_1_, a_1_a_2_, a_2_a_2_*, respectively:

$$Var(G_{j})_{ovdom}=2(1-p)p(1-F-2(-1+F)^{2}p+2(-1+F)^{2}p^{2})$$

$$cov\left(G_{i},G_{j}\right)_{ovdom}=-\frac{4F(-1+p)p((1-2p)^{2}+2F^{3}(-1+p)p+F(-1-6(-1+p)p))}{(1+F)(1+2F)}$$

Expressions for $Var\left(T\right)$ and $Var_{0}(T)$ are defined in (1.9) and (1.2) respectively.

Using (1.10), one can obtain values for variance inflation factor. In the future, for the sake of simplicity we assume:

$K=\sum^{ } (a_{k}-b_{k})^{2}$ (1.11)
