# Supplementary material for: Development and Application of Genomic Control Methods for Genome-Wide Association Studies Using Non-Additive Models
Source: PLoS One. 2013 Dec 16;8(12):e81431. doi: 10.1371/journal.pone.0081431 (PMC3864791; doi:10.1371/journal.pone.0081431)
Supplement: Table S1 — The distribution of genotypes of biallelic markers in the “case-control” design. (DOC) [file pone.0081431.s002.doc]

Table S1.The distribution of genotypes of biallelic markers in the "case-control" design.

|  | *a_1_a_1_* | *a_1_a_2_* | *a_2_a_2_* | Summary |
| --- | --- | --- | --- | --- |
| Cases | *r_0_* | *r_1_* | *r_2_* | *R* |
| Controls | *s_0_* | *s_1_* | *s_2_* | *S* |
| Summary | *m_0_* | *m_1_* | *m_2_* | *N* |
