# Supplementary material for: Development and Application of Genomic Control Methods for Genome-Wide Association Studies Using Non-Additive Models
Source: PLoS One. 2013 Dec 16;8(12):e81431. doi: 10.1371/journal.pone.0081431 (PMC3864791; doi:10.1371/journal.pone.0081431)
Supplement: Table S2 — Formalization of the 1df models of inheritance using different genotype coding . (DOC) [file pone.0081431.s003.doc]

**Table S2. Formalization of the 1df models of inheritance using different genotype coding** $t_{i}$.

|  | Model of inheritance | | | |
| --- | --- | --- | --- | --- |
| Genotype | Various | Recessive | Additive | Dominant |
| *a_1_a_1_* | 0 | 0 | 0 | 0 |
| *a_1_a_2_* | *x* | 0 | ½ | 1 |
| *a_2_a_2_* | 1 | 1 | 1 | 1 |
