# Supplementary material for: Development and Application of Genomic Control Methods for Genome-Wide Association Studies Using Non-Additive Models
Source: PLoS One. 2013 Dec 16;8(12):e81431. doi: 10.1371/journal.pone.0081431 (PMC3864791; doi:10.1371/journal.pone.0081431)
Supplement: Table S3 — Joint probability distribution of genotype frequencies. (DOC) [file pone.0081431.s004.doc]

Table S3. Joint probability distribution of genotype frequencies.

| G_1_,G_2_ | N | Pr(G_1_,G_2_)*(1+F)(1+2F) |
| --- | --- | --- |
| *a_2_a_2_, a_2_a_2_* | 1 | *6F^3^p + 11F^2^(1-F)p^2^ + 6F(1-F)^2^p^3^ + (1-F)^3^p^4^* |
| *a_2_a_2_, a_1_a_2_* | 4 | *2F^2^(1-F)pq + 3F(1-F)^2^p^2^q + (1-F)^3^p^3^q* |
| *a_2_a_2_, a_1_a_1_* | 2 | *F(1-F)pq + (1-F)^3^p^2^q^2^* |
| *a_1_a_2_, a_1_a_2_* | 4 | *F(1-F)pq + (1-F)^3^p^2^q^2^* |
| *a_1_a_1_, a_1_a_2_* | 4 | *2F^2^(1-F)pq + 3F(1-F)^2^pq^2^ + (1-F)^3^pq^3^* |
| *a_1_a_1_, a_1_a_1_* | 1 | *6F^3^q + 11F^2^(1-F)q^2^ + 6F(1-F)^2^q^3^ + (1-F)^3^q^4^* |
