# Supplementary material for: Development and Application of Genomic Control Methods for Genome-Wide Association Studies Using Non-Additive Models
Source: PLoS One. 2013 Dec 16;8(12):e81431. doi: 10.1371/journal.pone.0081431 (PMC3864791; doi:10.1371/journal.pone.0081431)
Supplement: Table S4 — Results of Levene's test for homogeneity of variance between two correction methods of E* (proportion of the tests with p-value≤0.05) - in simulations for type 1 error. Var1 and Var2 – total variance of E* in all frequency groups for the first and second method, respectively. Ratio – ratio of Var1 and Var2. (DOC) [file pone.0081431.s005.doc]

**Table S4. Results of Levene's test for homogeneity of variance between two correction methods of E* (proportion of the tests with p-value ≤ 0.05) - in simulations for type 1 error.** Var1 and Var2 – total variance of E* in all frequency groups for the first and second method, respectively. Ratio – ratio of Var1 and Var2.

| **Methods to compare** | **Model** | **Var1** | **Var2** | **Ratio** | **F-statistic** | **P-value** |
| --- | --- | --- | --- | --- | --- | --- |
| **Constant corrected and VIF** | Recessive | 7.34E-05 | 6.69E-06 | 10.97 | 5518.13 | 0.00E+00 |
|  | Additive | 6.76E-06 | 4.78E-06 | 1.41 | 131.05 | 1.95E-29 |
|  | Dominant | 7.13E-05 | 6.45E-06 | 11.06 | 5670.45 | 0.00E+00 |
|  | Over-dominant | 8.26E-05 | 5.94E-06 | 13.91 | 5398.39 | 0.00E+00 |
|  | Genotypic (df=2) | 7.72E-06 | 5.49E-06 | 1.41 | 105.57 | 3.62E-24 |
| **Constant corrected and PGC** | Recessive | 7.34E-05 | 3.59E-06 | 20.45 | 7106.93 | 0.00E+00 |
|  | Additive | 6.76E-06 | 1.85E-05 | 0.36 | 23.51 | 1.34E-06 |
|  | Dominant | 7.13E-05 | 3.54E-06 | 20.13 | 7244.72 | 0.00E+00 |
|  | Over-dominant | 8.26E-05 | 3.91E-06 | 21.12 | 6257.51 | 0.00E+00 |
|  | Genotypic (df=2) | 7.72E-06 | 3.49E-06 | 2.21 | 598.67 | 7.28E-116 |
| **PGC and VIF** | Recessive | 6.69E-06 | 3.59E-06 | 1.86 | 430.68 | 8.64E-87 |
|  | Additive | 4.78E-06 | 1.85E-05 | 0.26 | 0.56 | 4.54E-01 |
|  | Dominant | 6.45E-06 | 3.54E-06 | 1.82 | 415.04 | 5.56E-84 |
|  | Over-dominant | 5.94E-06 | 3.91E-06 | 1.52 | 178.38 | 4.91E-39 |
|  | Genotypic (df=2) | 5.49E-06 | 3.49E-06 | 1.57 | 229.22 | 4.19E-49 |
